# Global incidence and mortality of neonatal sepsis: a systematic review and meta-analysis

**DOI:** 10.1136/archdischild-2020-320217

**Published:** 2021-01-22

**Authors:** Carolin Fleischmann, Felix Reichert, Alessandro Cassini, Rosa Horner, Thomas Harder, Robby Markwart, Marc Tröndle, Yoanna Savova, Niranjan Kissoon, Peter Schlattmann, Konrad Reinhart, Benedetta Allegranzi, Tim Eckmanns

**Affiliations:** 1 Center for Sepsis Control and Care, Jena University Hospital, Jena, Germany; 2 Institute of Infectious Diseases and Infection Control, Jena University Hospital, Jena, Germany; 3 Department of Infectious Disease Epidemiology, Robert Koch Institute, Berlin, Berlin, Germany; 4 Postgraduate Training for Applied Epidemiology (PAE), Robert Koch Institute, Berlin, Germany; 5 European Programme for Intervention Epidemiology Training (EPIET), European Centre for Disease Prevention and Control (ECDC), Stockholm, Sweden; 6 Infection Prevention and Control Hub, Integrated Health Services, World Health Organization HQ, Geneva, GE, Switzerland; 7 Institute of General Practice and Family Medicine, Jena University Hospital, Jena, Germany; 8 University of British Columbia and British Columbia Children’s Hospital, Vancouver, British Columbia, Canada; 9 Institute for Medical Statistics, Computer Science and Data Science, Jena University Hospital, Jena, Germany; 10 Department of Anesthesiology and Operative Intensive Care Medicine (CCM, CVK), Charité Universitätsmedizin Berlin, corporate member of Freie Universität Berlin, Humboldt-Universität zu Berlin, Berlin, Germany

**Keywords:** neonatology, epidemiology, mortality

## Abstract

**Background:**

Neonates are at major risk of sepsis, but data on neonatal sepsis incidence are scarce. We aimed to assess the incidence and mortality of neonatal sepsis worldwide.

**Methods:**

We performed a systematic review and meta-analysis. 13 databases were searched for the period January 1979–May 2019, updating the search of a previous systematic review and extending it in order to increase data inputs from low-income and middle-income countries (LMICs). We included studies on the population-level neonatal sepsis incidence that used a clinical sepsis definition, such as the 2005 consensus definition, or relevant ICD codes. We performed a random-effects meta-analysis on neonatal sepsis incidence and mortality, stratified according to sepsis onset, birth weight, prematurity, study setting, WHO region and World Bank income level.

**Results:**

The search yielded 4737 publications, of which 26 were included. They accounted for 2 797 879 live births and 29 608 sepsis cases in 14 countries, most of which were middle-income countries. Random-effects estimator for neonatal sepsis incidence in the overall time frame was 2824 (95% CI 1892 to 4194) cases per 100 000 live births, of which an estimated 17.6% 9 (95% CI 10.3% to 28.6%) died. In the last decade (2009–2018), the incidence was 3930 (95% CI 1937 to 7812) per 100 000 live births based on four studies from LMICs. In the overall time frame, estimated incidence and mortality was higher in early-onset than late-onset neonatal sepsis cases. There was substantial between-study heterogeneity in all analyses. Studies were at moderate to high risk of bias.

**Conclusion:**

Neonatal sepsis is common and often fatal. Its incidence remains unknown in most countries and existing studies show marked heterogeneity, indicating the need to increase the number of epidemiological studies, harmonise neonatal sepsis definitions and improve the quality of research in this field. This can help to design and implement targeted interventions, which are urgently needed to reduce the high incidence of neonatal sepsis worldwide.

**What is already known on this topic?**

- The Global Burden of Disease (GBD) estimated 1.3 million annual incident cases of neonatal sepsis and other infections (approximately 937 cases per 100 000 live births) and 203 000 sepsis-attributable neonatal deaths. However, important contributors to the burden of neonatal infection and sepsis, such as pneumonia, are not captured by this estimate based on the GBD definition.
- A previous systematic review identified only eight studies on neonatal sepsis incidence from five countries that used a clinical sepsis definition.

**What this study adds?**

- We found in 26 studies a pooled neonatal sepsis incidence of 2824 sepsis cases per 100 000 live births (95% CI 1892 to 4194) and a mortality of 17.6% (95% CI 10.3% to 28.6%). Preterm and very low birthweight neonates were particularly affected, and there were considerable regional differences in incidence.
- Data are lacking from many countries, underlining the need for further epidemiological research to guide interventions that reduce neonatal incidence and mortality.

## Introduction

Sepsis is a dysregulated host response to infection leading to life-threatening organ dysfunction.^1^ Although neonates are highly vulnerable to sepsis, incidence estimates for this age group are lacking from many countries.^2^ Recently, the Global Burden of Disease (GBD) Study 2016/2017 estimated 1.3 (95% CI 0.8 to 2.3) million annual incident cases of neonatal sepsis worldwide,^3^ resulting in 203 000 (95% CI 178 700 to 267 100) sepsis-attributable deaths.^4^ Neonates are disproportionately affected in low-income and middle-income countries (LMICs) with a high prevalence of infectious diseases^5^ and poor access to adequately equipped and staffed healthcare facilities.^6^ In sub-Saharan Africa alone, an estimated 5.3–8.7 million disability-adjusted life-years have been lost in 2014 due to neonatal sepsis and consecutive long-term morbidity.^7^ Neonatal sepsis has resulted in an estimated economic burden of up to US$469 billion in this region (2014 data).^7^

In a previous systematic review and meta-analysis, we compiled evidence on the burden of paediatric sepsis including neonatal sepsis.^2^ Only eight studies that used a clinical definition of neonatal sepsis could be identified, of which five studies originated from LMICs. We aimed to assess the global incidence and mortality of neonatal sepsis with a particular focus on LMICs by updating and extending this previous systematic review. Secondary objectives were to compile data on underlying organisms and antimicrobial resistance, hospital length of stay and sepsis-attributable mortality.

## Methods

The protocol of this systematic review was registered in PROSPERO (CRD42020149085).

### Search strategy and selection criteria

As starting point, we used the previously published systematic review on neonatal sepsis incidence.^2^ All studies included in this review were added to our search. We then searched 13 electronic databases: MEDLINE, Embase, LILACS, African Journals Online, OpenGREY, MedCarib, Index Medicus for the WHO Eastern Mediterranean, African, South East Asia and Western Pacific Regions, IndMed, Web of Science and WHOLIS. No language or publication restrictions were applied. We updated the search strategy from the previous systematic review.^2^ Furthermore, we used a comprehensive search strategy that combined a list of sepsis terms with the individual names of LMICs suggested by the Cochrane Effective Practice and Organisation of Care (EPOC)^8^ group to search for studies published between January 1979 and May 2019 (see online supplmental appendix 1). Moreover, we hand-searched reference lists of relevant publications. Studies were eligible for inclusion if they reported population-level incidence or prevalence of neonatal sepsis (neonatal sepsis cases per live births of the hospital or region in a defined time frame). Neonatal sepsis had to be defined according to the International Consensus Conference on Paediatric Sepsis Definitions,^9^ sepsis 1,^10^ sepsis 2^11^ or sepsis 3^1^ or modified clinical criteria, such as clinical signs of neonatal sepsis (hypothermia, bradycardia and apnoea) in the presence of infection. Studies based on sepsis-relevant ICD-9/ICD-10 codes were also included. Since definitions varied widely, we accepted definitions of early-onset sepsis (EOS) as sepsis within the first 2–7 days of life. Depending on the EOS definition in each study, late-onset sepsis (LOS) was defined as sepsis diagnosed from 3 to 8 days until 28 days after birth, resulting in individual studies with no double counting, but in overlap between the definitions of EOS and LOS. We excluded studies that solely reported culture-proven sepsis cases, incidence per hospital admissions, or used the definition of possible severe bacterial infections (PSBIs) instead of neonatal sepsis, since neonatal sepsis accounts for only one out of four cases of PSBI.^12^ We excluded studies limited to other subgroups of sepsis, pathogens or specific patient groups, and studies with a missing study methodology description.

10.1136/archdischild-2020-320217.supp2 Supplementary data

### Data compilation and risk of bias assessment

Abstracts and full texts were reviewed by two independent investigators (CF, FR, MT, RH and YS). Discrepancies were resolved by discussion. Non-English articles were assessed by native speakers with medical backgrounds. Data extraction items and strategies can be found in the online supplemental appendix. Pathogen and antibiotic resistance data were only considered from the year 2000 onwards. Authors were contacted to specify methods and to provide additional data. Studies that met the inclusion criteria were assessed for risk of bias by the Hoy tool.^13^

### Statistical analyses

We categorised the studies by sepsis type (EOS, LOS and EOS/LOS combined). For subgroup analyses, participants were stratified by birth weight into normal birth weight (≥2500 g), low birth weight (1500–2499 g) and very low birth weight (<1500 g) neonates, and by gestational age into preterm (<37 weeks) and term neonates. Countries were categorised by WHO region and World Bank income level, and studies by community-based or hospital-based design and decade in which the study was conducted according to the start of their observation period (before 1989, 1989–1998, 1999–2008 and 2009–2018). We conducted meta-analyses of sepsis incidence per 100 000 live births, mortality per 100 sepsis cases and the proportion of culture-proven sepsis with 95% CI using the package ‘meta’ V.4.9.5 in R V.3.6.1 (online supplemental appendix 2). We calculated pooled estimates using a random-effects model with variance stabilising logit transformed proportions and estimated between-study variance τ² using the Sidik-Jonkman estimator. We used I² statistics to quantify statistical heterogeneity.

## Results

**Figure 1:**
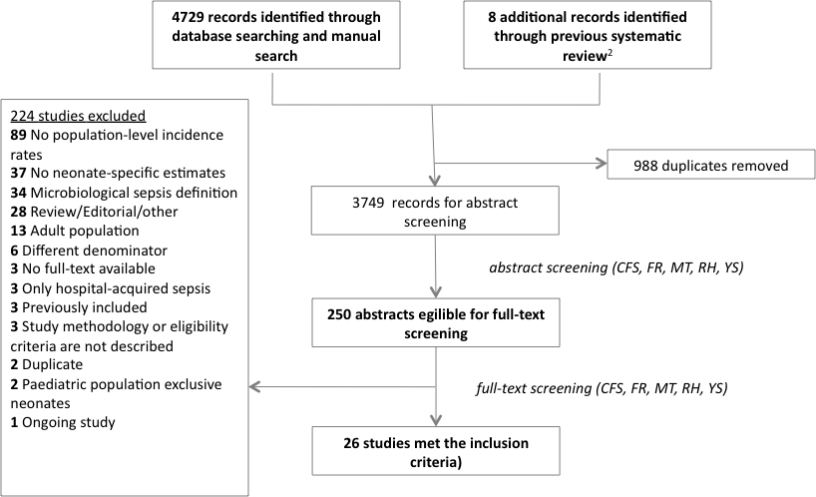
Flow of study inclusion.

We identified 4737 records (figure 1); 250 studies underwent full-text screening; and 26 fulfilled the inclusion criteria.^14–39^

**Figure 2:**
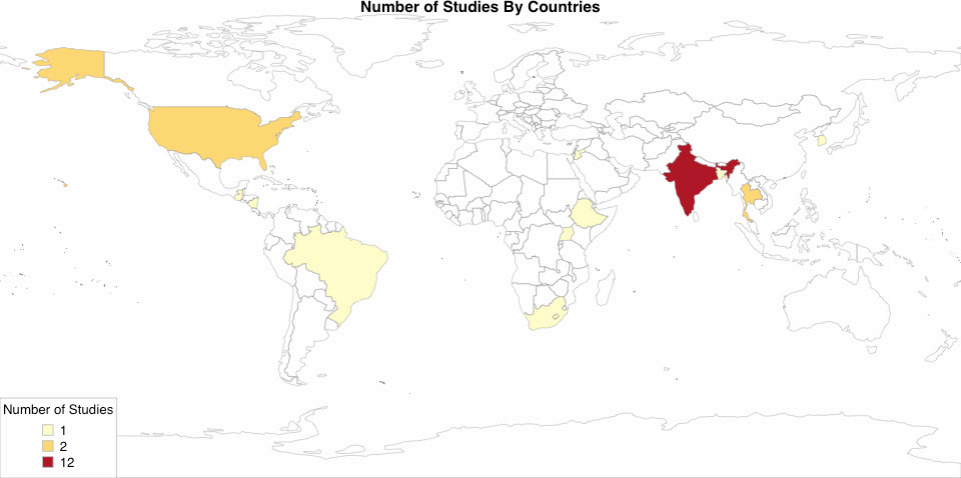
Number of studies on neonatal sepsis incidence (early-onset and late-onset sepsis combined) included per country.

The entire study population comprised 2 797 879 live births in 14 countries and five WHO regions (online supplemental appendix eTable 1 and figure 2). Two studies originated from low-income countries, 20 from middle-income and four from high-income countries (HICs). Studies were mostly prospective (n=18/26). We included 21 cohort studies, 4 trials and 1 case–control study. The majority were single-centre studies. Seven studies used a community-based design; 19 were hospital-based, including studies limited to neonatal intensive care units (NICUs) or studies with entire hospitals under observation. Studies were at moderate to high risk of bias (online supplemental appendix eTable 2).

Overall, 29 608 sepsis cases were identified. Sepsis definitions varied by diagnostic criteria and time intervals (online supplemental appendix Table e1).

Incidence of EOS/LOS combined was reported by 21 studies, of which 5 provided separate results for EOS and LOS. EOS and LOS were distinct subgroups in these studies with no overlap. The remaining five studies reported exclusively on EOS. If not stated otherwise, our results refer to studies that report on EOS/LOS combined. Separate analyses for EOS or LOS are provided if the number of studies was sufficient.

**Figure 3:**
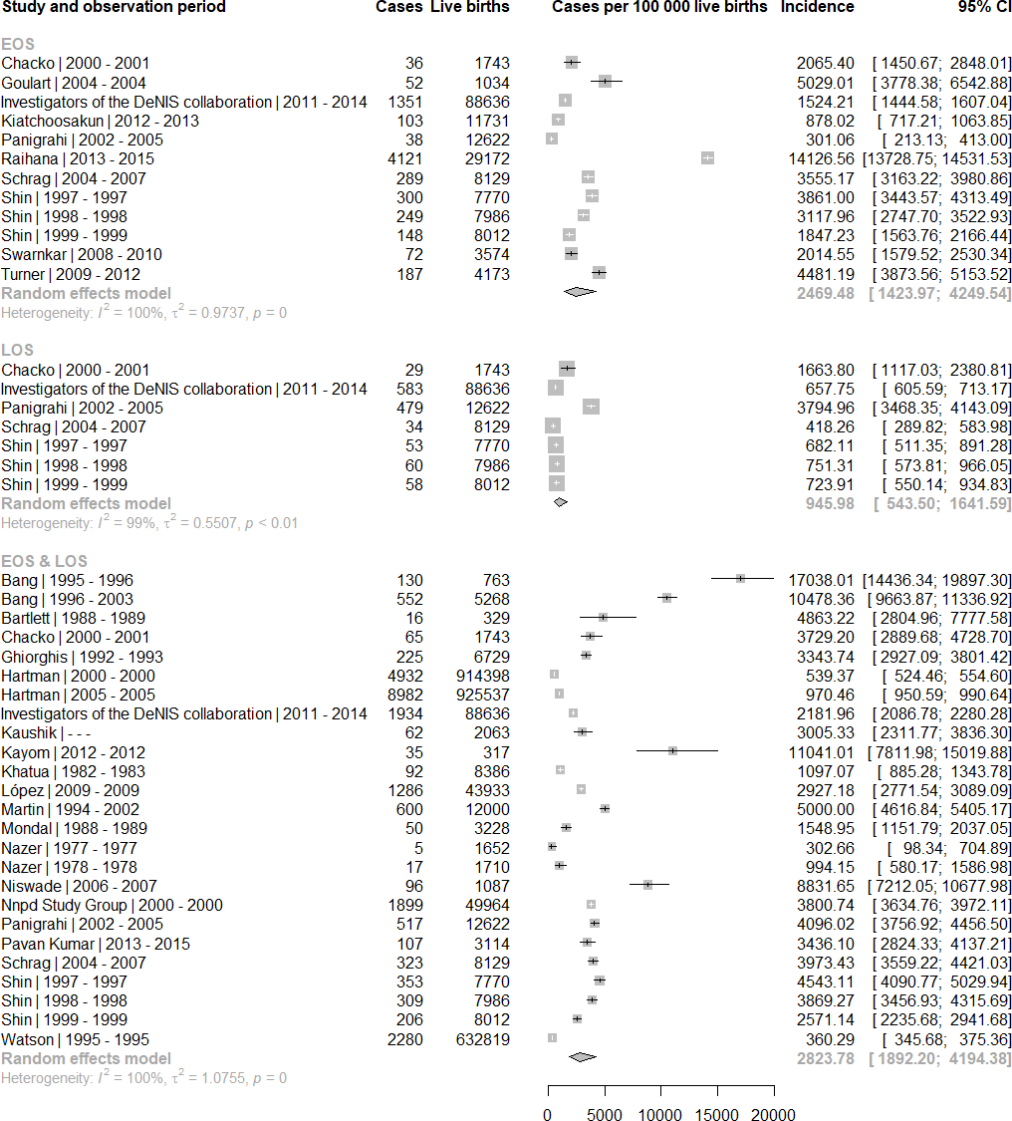
Incidence of neonatal sepsis (EOS, late-onset sepsis, and EOS/LOS combined) per 100 000 live births by sepsis type. EOS, early-onset sepsis; LOS, late-onset sepsis.

We found a random-effects estimate of 2824 neonatal sepsis cases per 100 000 live births (95% CI 1892 to 4194) in the overall time frame (before 1989–2018), with a 2.6 times higher incidence of EOS (2469/100 000, 95% CI 1424 to 4250) than LOS (946/100 000, 95% CI 544 to 1642) (figure 3). For the past decade (2009–2018), the random-effects estimate for EOS/LOS incidence was 3930/100 000 (95% CI 1937 to 7812) based on four studies from LMICs. Compared with estimates from past decades, we found no clear temporal trends but wide CI for each epoch (1999–2008: 2706, 95% CI 1451 to 4993; 1989–1998: 4012, 95% CI: 1808 to 8665; before 1989: 1250, 95% CI 534 to 2896). Of note, studies included in each time stratum differed in terms of study design and country of origin. The estimated incidence of EOS for the decade until 2018 was 3112/100 000 (95% CI 898 to 10 222) based on four studies (table 1). Only one study reported LOS incidence estimates from the past decade (investigators of Delhi Neonatal Infection Study (DeNIS) collaboration: 658, 95% CI 607 to 713, per 100 000).

**Table 1:** Meta-analysis estimates and heterogeneity

Sepsis type	Subgroup	Incidence estimates (n)	MA: incidence Random-effects estimates (cases per 100 000 live births)	I² (%)
By date				
EOS+LOS	Before 1989	5	1250.0 (534.4 to 2895.7)	90.9
	1989–1998	8	4012.4 (1808.5 to 8665.4)	99.9
	1999–2008	8	2706.3 (1450.9 to 4993.0)	99.9
	2009–2018	4	3930.4 (1936.9 to 7812.1)	98.1
EOS	Before 1989	–	–	–
	1989–1998	2	3476.0 (2841.9 to 4245.3)	84.5
	1999–2008	6	1878.2 (863.7 to 4035.7)	98.0
	2009–2018	4	3112.3 (898.2 to 10 222.1)	99.9
LOS	Before 1989	–	–	–
	1989–1998	2	Singe-study estimate: 718 (595.7 to 865.1)	0.0
	1999–2008	4	1189.2 (458.0 to 3052.0)	99.0
	2009–2018	1	Single-study estimate: 657.8 (606.6 to 713.2)	–
By patient subgroup				
EOS+LOS	NBW	2	2430.1 (1164.4 to 5001.7)	92.5
	LBW	2	7475.1 (5857.0 to 9495.1)	11.1
	VLBW	1	Single-study estimate: 31 428.6 (18 335.2 to 48 337.7)	–
	Term	1	Single-study estimate: 3593.4 (3202.9 to 4029.5)	–
	Preterm	1	Single-study estimate: 13 592.2 (10 202.5 to 17 883.9)	–
EOS	NBW	3	818.1 (204.5 to 3213.9)	96.6
	LBW	3	5421.5 (3706.5 to 7865.0)	76.8
	VLBW	3	17 128.8 (9192.1 to 29 678.5)	74.7
	Term	3	821.3 (200.5 to 3300.2)	97.1
	Preterm	3	10 251.7 (7890.8 to 13 217.7)	57.2
LOS	NBW	0	–	–
	LBW	1	Single-study estimate: 517.2 (166.9 to 1591.1)	–
	VLBW	1	Single-study estimate: 1388.9 (86.3 to 18 672.7)	–
	Term	0	–	–
	Preterm	1	Single-study estimate: 323.6 (45.6 to 2260.0)	–
By WHO region				
EOS+LOS	AFR	3	5243.6 (2504.6 to 10 650.6)	95.5
	PAR	6	1541.6 (613.5 to 3819.8)	99.9
	SEAR	11	4005.1 (2438.5 to 6511.2)	99.4
	EMR	2	585.5 (192.8 to 1764.1)	81.8
	WPR	3	3573.9 (2570.8 to 4948.6)	95.5
EOS	AFR	1	Single-study estimate: 3555.2 (3173.9 to 3980.4)	–
	PAR	1	Single-study estimate: 5029.0 (3852.2; 6540.9)	–
	SEAR	7	1991.8 (795.0 to 4901.0)	99.9
	EMR	0	–	–
	WPR	3	2824.4(1843.9; 4303.6)	96.4
LOS			NA	
By World Bank income level				
EOS+LOS	Low	2	6086.4 (1843.1 to 18 280.0)	97.8
	Middle	16	3195.3 (1991.2 to 5089.7)	99.2
	High	7	1722.3 (769.2 to 3810.7)	99.9
EOS	Low	0	–	–
	Middle	9	2359.3 (1131.6 to 4853.6)	99.9
	High	3	2824.4 (1843.9 to 4303.6)	96.4
LOS			NA	
By study setting				
EOS+LOS	Community-based	6	8549.3 (5520.3; 13 011.5)	98.7
	Hospital-based	19	1986.5 (1350.0 to 2914.1)	99.9
EOS	Community-based	2	2187.2(44.6; 52 848.4)	99.8
	Hospital-based	10	2518.7 (1792.1 to 3529.5)	98.5
LOS			NA	

AFR, African Region; EMR, Eastern Mediterranean Region; EOS, early-onset sepsis; LBW, low birth weight; LOS, late-onset sepsis; MA, meta-analysis; NA, not applicable; NBW, normal birth weight; PAR, Region of the Americas; SEAR, South-East Asia Region; VLBW, very low birth weight; WPR, Western Pacific Region.

Birth weight and gestational age were inversely related to EOS incidence, resulting in the highest incidence in VLBW (17 129/100 000, 95% CI 9192 to 29 679) and preterm neonates (10 252/100 000, 95% CI 7891 to 13 218) (online supplemental appendix eFigure 1).

Incidence estimates were higher in community-based studies (8549/100 000, 95% CI 5520 to 13 011) than in hospital-based studies (1986/100 000, 95% CI 1350 to 2914) (online supplemental appendix eFigure 2). Although the 95% CIs for each income stratum were overlapping, compared with HICs, neonatal sepsis incidence was 1.8-fold higher in middle-income countries and 3.5-fold higher in low-income countries (online supplemental appendix figure 3). Incidence was highest in studies from Africa (5244/100 000, 95% CI 2505 to 10 651; table 1).

We observed substantial heterogeneity between studies, even within subgroup analyses.

Overall, the random-effects estimate for neonatal sepsis case mortality was 17.6% (95% CI 10.3% to 28.6%), and 16.4% (95% CI 9.8 to 26.1) and 9.1% (95% CI 2.1 to 32.5) for EOS and LOS, respectively (online supplemental appendix figure 4). Again we observed substantial heterogeneity (I²≥90%). The investigators of DeNIS collaboration attributed 24% of neonatal deaths to sepsis.^18^

In total, 16 studies reported a proportion of culture-proven sepsis, resulting in a random-effects estimate of 31.8% (95% CI 23.5% to 41.4%) for EOS/LOS combined (online supplemental appendix figure 5). For EOS, lower proportions were reported (11.4%, 95% CI 2.7 to 37.1%) based on six studies than for LOS (27.5%, 95% CI 8.9% to 59.5%) based on three studies. Most commonly identified pathogens were *Staphylococcus aureus* and *Klebsiella* spp (online supplemental appendix table 3). Results on antimicrobial resistance, which was reported in 3 out of 16 studies, are presented in online supplemental appendix table 3.

## Discussion

Our estimates confirm that neonatal sepsis is an important contributor to neonatal morbidity worldwide, with a higher burden in LMICs. Based on the available evidence of 26 studies from 14 countries, we found an incidence of 2824 neonatal sepsis cases per 100 000 live births in the overall time frame. Mortality was 17.6%. The incidence estimate was 1.4-fold higher in the past decade (2009–2018, 3930/100 000 live births), but this estimate was exclusively relying on LMIC data, which might be one reason for the higher incidence. No recent data from HIC could be identified, indicating gaps in epidemiological research on neonatal sepsis all over the world. The overall incidence and mortality for neonatal sepsis was higher than the estimates for EOS and LOS. This could be explained by inclusion of different studies in the three meta-analyses. Incidence of EOS was higher in risk groups, such as LBW, VLBW and preterm infants. In our analyses, the observed population-level incidence of neonatal sepsis was more than four times higher in community-based compared with hospital-based studies. This may reflect high rates of mothers delivering outside a healthcare facility without a skilled birth attendant, unsterile cord care practices, reduced access to healthcare facilities and lower care seeking behaviours^40^ in LMICs, where the included community-based studies originated from. On the other hand, hospitalisation, improved survival of preterm newborns and use of invasive devices increase the incidence of nosocomial LOS cases, especially in HIC.^41 42^

Our most recent results of neonatal sepsis incidence in the past decade (2009–2018) indicate an approximately four times higher number of global neonatal sepsis cases than the GBD estimates (3930 vs 937 cases per 100 000), likely due to different methodologies, such as varying case definitions, data collection methods and data inputs from different countries. Extrapolations from our findings must be interpreted with caution since the included studies may not be representative of the global population and carry a moderate to high risk of bias. Contrary to most studies that we included in our review, cases of pneumonia are captured in the GBD modelling of pneumonia as a separate entity, but not as neonatal sepsis cases,^3^ which is a major methodological difference. Furthermore, the GBD study used more recent data to compute estimates for the year 2017, while our most recent estimates refer to the past decade (2009–2018).^3^ The recent GBD study, which used another methodology to assess sepsis-associated deaths and hospital incidence of sepsis, estimated 20.3 million incident sepsis cases in children aged <5 years per year.^43^ The authors did not present specific data on neonatal sepsis. However, given that neonates account for approximately two out of three sepsis cases in children aged <5 years,^17^ the estimated incidence is likely higher than those in previous GBD studies.

Approximately one-third of infections were culture-proven with *S. aureus* and *Klebsiella* spp as the most common causative pathogens. However, in most studies that reported aetiology, the assessments of causative pathogens were based on only a low number of isolates or originated from single-centre studies. A cautious interpretation of these results is therefore necessary, other systematic reviews with a scope on aetiology and antibiotic susceptibility could compile more evidence.^44 45^ Susceptibility testing results were reported too seldomly and may be outdated, and the data are too heterogeneous to draw conclusions.

The extended search strategy identified 17 additional studies from LMICs and 1 additional study from an HIC. In general, systematic reviews that lack information from LMICs may benefit from a broader search strategy but inclusion of specific LMIC country names, and filters for LMIC as proposed by the EPOC group. We included data from seven community-based studies that followed up a large number of newborns in partly rural regions by trained village health workers/teams and are of particular value to better understand the neonatal sepsis epidemiology in remote and rural community settings in low-income countries.

Major knowledge gaps on the population-based epidemiology of neonatal sepsis remain in most countries, particularly, but not exclusively, in LMICs. Reasons for this may be the lack of a robust research infrastructure, formal healthcare systems^46^ or prioritisation of other important healthcare issues. Furthermore, several large observational studies that target severe neonatal infections did not meet our inclusion criteria either due of their scope or the inclusion criteria.

There are limitations of our study. First, the studies we included may not be representative of the global population for several reasons. Data inputs were from 14 countries from five WHO regions; thus information on the majority of countries and the European WHO region was not included. Numerous studies only reported the incidence of NICU-treated or hospital-treated neonatal sepsis, which may underestimate the incidence of neonatal sepsis by excluding diseased infants outside the hospital setting. This is of particular relevance in LMICs, where a considerable number of births occur outside the hospital^47^ and healthcare seeking for sick neonates may be low.^40^ Furthermore, most hospital-based studies were performed in tertiary care hospitals, which deliver higher quality of care compared with community hospitals, where a large number of neonates may seek care in some contexts. Second, we cannot judge on temporal trends as the number of studies in the respective time strata was low. Thus, differences in incidence estimates depending on the years of observation may be also explained by the fact that studies originated from different regions and used study designs with limited comparability. Third, the included studies were at moderate to high risk of bias, mainly due to the aforementioned lack of representativeness and a reliable case definition. Fourth, we observed high between-study heterogeneity, possibly due to differing study designs, settings and observation periods. The pooled estimates include population-level estimates from community-based studies, as well as NICU-based and hospital-based studies from facilities of different levels of care. General access, capacities and admission policies may differ between NICUs and countries. Heterogeneity may also be driven by different definitions. Applied sepsis definitions are mostly adaptations of the clinical consensus criteria,^9^ such as when laboratory testing was unavailable,^31^ or relied on selected clinical symptoms of neonatal sepsis. Currently, there is no sepsis definition for the neonatal age group in line with the current sepsis definition,^48^ which can be applied in all resource settings and also in preterm neonates. That leads to wide variations of existing neonatal sepsis definitions^49^ and hampers the comparability of sepsis epidemiology studies. In our review, we accepted a wide range of definitions, including those based on clinical criteria, to not exclude studies from LMICs. We pooled estimates from all included studies to gather all available evidence on the epidemiology of neonatal sepsis. Fifth, the underlying causes of death in these neonates were not reported except in one of the included studies, which concluded a sepsis-attributable mortality rate of 24%. The remaining studies referred to the proportion of patients with neonatal sepsis who died, but the cause of death was not specified. Finally, while our systematic review and pooled estimates consider only live births, globally, one out of four stillbirths are attributed to infection,^50^ adding substantially to the overall burden of perinatal deaths that are due to sepsis.

The limitations identified by our study highlight the need to increase and improve epidemiological research on neonatal sepsis. A crucial prerequisite is the establishment of a harmonised definition and validated diagnostic criteria for neonatal sepsis applicable in all resource settings and in preterm neonates. Furthermore, a standardisation of study designs and reporting, for example, in line with the Strengthening the Reporting of Observational Studies in Epidemiology for Newborn Infection checklist, is important.^51^ Further investments in epidemiological research infrastructure and capacity in both LMICs and HICs are needed in order to improve surveillance of neonatal sepsis, particularly in community-based designs. Having robust methodologies to measure the burden of neonatal sepsis will be extremely useful to assess the effect of urgently needed interventions to prevent it. Indeed, a lot needs to be done yet to prevent infections in neonates and to reduce the frequency of their evolution to sepsis complications. The WHO issued recommendations on key prevention and control measures both in the community and hospital setting and improved early recognition and timely and appropriate treatment of neonatal sepsis, as well as recommendations for early essential newborn care.^52–55^ This includes, among others, exclusive breast feeding, skin-to-skin contact with the mother from birth, the empirical antibiotic treatment of neonates with signs of severe infection/sepsis (eg, fast breathing) and education of families on the recognition of signs of neonatal sepsis by trained community health workers.^54 55^ Given that approximately one quarter of neonatal deaths is related to infection and sepsis, progress in reducing the burden of neonatal sepsis is critical to reduce global child mortality and of high public health relevance.

10.1136/archdischild-2020-320217.supp1 Supplementary data

## Data Availability

Data are available upon reasonable request. The datasets analysed during the current study are available from the corresponding author on reasonable request. The software codes used during the current study are available in online supplemental file 2.
