# Supplementary material for: Global incidence and mortality of neonatal sepsis: a systematic review and meta-analysis
Source: Arch Dis Child. 2021 Jan 22;106(8):745–52. doi: 10.1136/archdischild-2020-320217 (PMC8311109; doi:10.1136/archdischild-2020-320217)
Supplement: Supplementary data [file archdischild-2020-320217supp002.pdf]

Supplement

Global incidence and mortality of neonatal sepsis: systematic review and meta-analysis

Fleischmann-Struzek C, MD*[1], Reichert F, MD*[2,3,4], Cassini A, MD[5], Horner R[2], Harder T, MD[2], Markwart R, PhD[2], Troendle M[2], Savova Y[2], Prof. Kissoon N, MD[6], Prof. Schlattmann P, MD[7], Prof. Reinhart K, MD[8,9,10], Prof. Allegranzi B, MD[5], Eckmanns T, MD[2]

*contributed equally

1 Center for Sepsis Control and Care, Jena University Hospital, Jena, Germany

2 Department of Infectious Disease Epidemiology, Robert Koch Institute, Berlin, Germany

3 Postgraduate Training for Applied Epidemiology (PAE), Robert Koch Institute, Berlin, Germany

4 European Programme for Intervention Epidemiology Training (EPIET), European Centre for Disease Prevention and Control (ECDC), Stockholm, Sweden

5 Infection Prevention and Control Hub, Integrated Health Services, World Health Organization HQ, Geneva, Switzerland

6 University of British Columbia and British Columbia Children's Hospital, Vancouver, BC, Canada

7 Institute for Medical Statistics, Computer Science and Data Science, Jena University Hospital, Jena, Germany

8 Department of Anesthesiology and Operative Intensive Care Medicine (CCM, CVK), Charité Universitätsmedizin Berlin, corporate member of Freie Universität Berlin, Humboldt-Universität zu Berlin, Berlin, Germany

9 Berlin Institute of Health, Campus Virchow-Klinikum, Berlin, Germany

10 Institute of Infectious Diseases and Infection Control, Jena University Hospital, Jena, Germany

This supplement forms part of the original submission.

We post it as supplied by the authors.

BMJ Publishing Group Limited (BMJ) disclaims all liability and responsibility arising from any reliance placed on this supplemental material which has been supplied by the author(s)

Supplement: Search strategy

Pubmed (and adapted for all other databases)

(((sepsis[Title] OR septic*[Title]) AND (Developing Countries*[Title/Abstract] OR Africa*[Title/Abstract] OR Asia*[Title/Abstract] OR Caribbean[Title/Abstract] OR West Ind*[Title/Abstract] OR South America*[Title/Abstract] OR Latin America*[Title/Abstract] OR Central America*[Title/Abstract] OR Afghanistan* OR Albania*[Title/Abstract] OR Algeria*[Title/Abstract] OR Angola*[Title/Abstract] OR Antigua*[Title/Abstract] OR Barbuda*[Title/Abstract] OR Argentina*[Title/Abstract] OR Armenia[Title/Abstract] OR Armenian[Title/Abstract] OR Aruba*[Title/Abstract] OR Azerbaijan*[Title/Abstract] OR Bahrain*[Title/Abstract] OR Bangladesh*[Title/Abstract] OR Barbados*[Title/Abstract] OR Benin*[Title/Abstract] OR Byelarus[Title/Abstract] OR Byelorussian[Title/Abstract] OR Belarus*[Title/Abstract] OR Belorussian[Title/Abstract] OR Belorussia[Title/Abstract] OR Beliz*[Title/Abstract] OR Bhutan*[Title/Abstract] OR Bolivia*[Title/Abstract] OR Bosnia*[Title/Abstract] OR Herzegovina*[Title/Abstract] OR Hercegovina*[Title/Abstract] OR Botswana*[Title/Abstract] OR Brasil*[Title/Abstract] OR Brazil*[Title/Abstract] OR Bulgaria*[Title/Abstract] OR Burkina Faso*[Title/Abstract] OR Burkina Fasso*[Title/Abstract] OR Upper Volta*[Title/Abstract] OR Burundi*[Title/Abstract] OR Urundi*[Title/Abstract] OR Cambodia*[Title/Abstract] OR Khmer Republic*[Title/Abstract] OR Kampuchea*[Title/Abstract] OR Cameroon*[Title/Abstract] OR Cameroons*[Title/Abstract] OR Cameron*[Title/Abstract] OR Cape Verde[Title/Abstract] OR Central African Republic*[Title/Abstract] OR Chad*[Title/Abstract] OR Chile*[Title/Abstract] OR China*[Title/Abstract] OR Colombia*[Title/Abstract] OR Comoros*[Title/Abstract] OR Comoro Island*[Title/Abstract] OR Comores[Title/Abstract] OR Mayotte[Title/Abstract] OR Congo*[Title/Abstract] OR Zaire*[Title/Abstract] OR Costa Rica*[Title/Abstract] OR Cote d'Ivoire[Title/Abstract] OR Ivory Coast[Title/Abstract] OR Croatia*[Title/Abstract] OR Cuba*[Title/Abstract] OR Cyprus*[Title/Abstract] OR Czechoslovakia*[Title/Abstract] OR Czech Republic*[Title/Abstract] OR Czechia*[Title/Abstract] OR Slovakia*[Title/Abstract] OR Slovak Republic*[Title/Abstract] OR Djibouti*[Title/Abstract] OR French Somaliland*[Title/Abstract] OR Dominica*[Title/Abstract] OR Dominican Republic*[Title/Abstract] OR East Timor*[Title/Abstract] OR East Timur*[Title/Abstract] OR Timor Leste*[Title/Abstract] OR Ecuador*[Title/Abstract] OR Egypt*[Title/Abstract] OR United Arab Republic*[Title/Abstract] OR El Salvador*[Title/Abstract] OR Eritrea*[Title/Abstract] OR

Estonia*[Title/Abstract] OR Ethiopia*[Title/Abstract] OR Fiji*[Title/Abstract] OR Gabon*[Title/Abstract] OR Gabonese Republic[Title/Abstract] OR Gambia*[Title/Abstract] OR Gaza*[Title/Abstract] OR Georgia Republic*[Title/Abstract] OR Georgian Republic*[Title/Abstract] OR Ghana*[Title/Abstract] OR Gold Coast*[Title/Abstract] OR Greece[Title/Abstract] OR Grenada*[Title/Abstract] OR Guatemala*[Title/Abstract] OR Guinea*[Title/Abstract] OR Guam*[Title/Abstract] OR Guiana*[Title/Abstract] OR Guyana*[Title/Abstract] OR Haiti*[Title/Abstract] OR Honduras*[Title/Abstract] OR Hungar*[Title/Abstract] OR India OR Maldiv*[Title/Abstract] OR Indonesia*[Title/Abstract] OR Iran*[Title/Abstract] OR Iraq*[Title/Abstract] OR Isle of Man[Title/Abstract] OR Jamaica*[Title/Abstract] OR Jordan*[Title/Abstract] OR Kazakhstan*[Title/Abstract] OR Kazakh*[Title/Abstract] OR Kenya*[Title/Abstract] OR Kiribati*[Title/Abstract] OR Korea*[Title/Abstract] OR Kosovo*[Title/Abstract] OR Kyrgyzstan*[Title/Abstract] OR Kirghizia*[Title/Abstract] OR Kyrgyz Republic*[Title/Abstract] OR Kirghiz*[Title/Abstract] OR Kirgizstan*[Title/Abstract] OR Lao PDR*[Title/Abstract] OR Laos*[Title/Abstract] OR Latvia*[Title/Abstract] OR Lebanon*[Title/Abstract] OR Lesotho*[Title/Abstract] OR Basutoland*[Title/Abstract] OR Liberia*[Title/Abstract] OR Libya*[Title/Abstract] OR Lithuania*[Title/Abstract] OR Macedonia*[Title/Abstract] OR Madagascar*[Title/Abstract] OR Malagasy Republic*[Title/Abstract] OR Malaysia*[Title/Abstract] OR Malaya*[Title/Abstract] OR Malay*[Title/Abstract] OR Sabah*[Title/Abstract] OR Sarawak*[Title/Abstract] OR Malawi*[Title/Abstract] OR Nyasaland OR Mali*[Title/Abstract] OR Malta*[Title/Abstract] OR Marshall Island*[Title/Abstract] OR Mauritania*[Title/Abstract] OR Mauritius*[Title/Abstract] OR Agalega Island*[Title/Abstract] OR Mexico*[Title/Abstract] OR Micronesia*[Title/Abstract] OR Middle East*[Title/Abstract] OR Moldova*[Title/Abstract] OR Moldovia*[Title/Abstract] OR Moldovian*[Title/Abstract] OR Mongolia*[Title/Abstract] OR Montenegro*[Title/Abstract] OR Morocco*[Title/Abstract] OR Ifni*[Title/Abstract] OR Mozambique*[Title/Abstract] OR Myanmar*[Title/Abstract] OR Myanma*[Title/Abstract] OR Burma*[Title/Abstract] OR Namibia*[Title/Abstract] OR Nepal*[Title/Abstract] OR Netherlands Antilles[Title/Abstract] OR New Caledonia*[Title/Abstract] OR Nicaragua*[Title/Abstract] OR Niger*[Title/Abstract] OR Nigeria*[Title/Abstract] OR Northern Mariana Island*[Title/Abstract] OR Oman*[Title/Abstract] OR Muscat OR Pakistan*[Title/Abstract] OR Palau*[Title/Abstract] OR Palestine*[Title/Abstract] OR Panama*[Title/Abstract] OR Paraguay*[Title/Abstract] OR Peru*[Title/Abstract] OR Philippines*[Title/Abstract] OR Philipines*[Title/Abstract] OR Phillipines*[Title/Abstract]

BMJ Publishing Group Limited (BMJ) disclaims all liability and responsibility arising from any reliance placed on this supplemental material which has been supplied by the author(s)

OR Phillippines*[Title/Abstract] OR Poland*[Title/Abstract] OR Portugal*[Title/Abstract] OR Puerto Rico*[Title/Abstract] OR Romania*[Title/Abstract] OR Rumania*[Title/Abstract] OR Roumania*[Title/Abstract] OR Russia*[Title/Abstract] OR Russian*[Title/Abstract] OR Rwanda*[Title/Abstract] OR Ruanda*[Title/Abstract] OR Saint Kitts*[Title/Abstract] OR St Kitts*[Title/Abstract] OR Nevis*[Title/Abstract] OR Saint Lucia*[Title/Abstract] OR St Lucia*[Title/Abstract] OR Saint Vincent*[Title/Abstract] OR St Vincent*[Title/Abstract] OR Grenadines*[Title/Abstract] OR Samoa*[Title/Abstract] OR Samoan Island*[Title/Abstract] OR Navigator Island*[Title/Abstract] OR Navigator Island*[Title/Abstract] OR Sao Tome*[Title/Abstract] OR Saudi Arabia*[Title/Abstract] OR Senegal*[Title/Abstract] OR Serbia*[Title/Abstract] OR Montenegro*[Title/Abstract] OR Seychelles*[Title/Abstract] OR Sierra Leone*[Title/Abstract] OR Slovenia*[Title/Abstract] OR Sri Lanka*[Title/Abstract] OR Ceylon*[Title/Abstract] OR Solomon Island*[Title/Abstract] OR Somalia*[Title/Abstract] OR South Africa*[Title/Abstract] OR Sudan*[Title/Abstract] OR Suriname*[Title/Abstract] OR Surinam*[Title/Abstract] OR Swaziland*[Title/Abstract] OR eSwatini*[Title/Abstract] OR Syria*[Title/Abstract] OR Tajikistan*[Title/Abstract] OR Tadzhikistan*[Title/Abstract] OR Tadjikistan*[Title/Abstract] OR Tadzhik*[Title/Abstract] OR Tanzania*[Title/Abstract] OR Thailand*[Title/Abstract] OR Togo*[Title/Abstract] OR Togolese Republic*[Title/Abstract] OR Tonga*[Title/Abstract] OR Trinidad*[Title/Abstract] OR Tobago*[Title/Abstract] OR Tunisia*[Title/Abstract] OR Turk*[Title/Abstract] OR Turkmenistan*[Title/Abstract] OR Turkmen*[Title/Abstract] OR Uganda*[Title/Abstract] OR Ukrain*[Title/Abstract] OR Uruguay*[Title/Abstract] OR USSR*[Title/Abstract] OR Soviet Union*[Title/Abstract] OR Union of Soviet Socialist Republics*[Title/Abstract] OR Uzbekistan*[Title/Abstract] OR Uzbek*[Title/Abstract] OR Vanuatu*[Title/Abstract] OR New Hebrides*[Title/Abstract] OR Venezuela*[Title/Abstract] OR Vietnam*[Title/Abstract] OR Viet Nam*[Title/Abstract] OR West Bank*[Title/Abstract] OR Yemen*[Title/Abstract] OR Yugoslavia*[Title/Abstract] OR Zambia*[Title/Abstract] OR Zimbabw*[Title/Abstract] OR Rhodesia*[Title/Abstract] OR Cook Island*[Title/Abstract] OR Marshall Island*[Title/Abstract] OR Nauru*[Title/Abstract] OR Niue*[Title/Abstract] OR Papua New Guinea*[Title/Abstract] OR Tuvalu*[Title/Abstract] OR Vanuatu*[Title/Abstract]) AND ("1979/01/01"[PDat] : "3000/12/31"[PDat])) OR ((sepsis[Title] OR septic*[Title]) AND (epidemiolog*[Title] OR incidence[Title] OR burden[Title] OR prevalence[Title]) AND ("2016/05/01"[PDat] : "3000/12/31"[PDat]))) NOT "animals"[MeSH:noexp]

Supplement: Data extraction strategy

Extracted data included: Author, country, years of observation, study design and setting, sepsis definition, patient population (inborn/outborn, relevant risk groups [low birth weight (LBW), very low birth weight neonates (VLBW), preterm neonates]), exclusion criteria, live births, sepsis cases, population denominator, sepsis incidence, prevalence, mortality (proportion of neonatal sepsis patients that died), mortality per 100 000 live births, sepsis-attributable mortality, hospital length of stay, underlying infections, proportion of culture-proven sepsis, isolated pathogens, and antimicrobial resistance.

If results were reported by year, respective years were extracted separately. If only the number of inborn live births was known, sepsis cases within this population were extracted. Trial data were extracted if interventions did not target sepsis or were only from observation periods prior to the intervention. For nationwide studies with missing population denominator, the number of live births was extracted from national census registries.

Supplement eFig. 1: Incidence of early-onset sepsis per 100 000 livebirths by sub-group

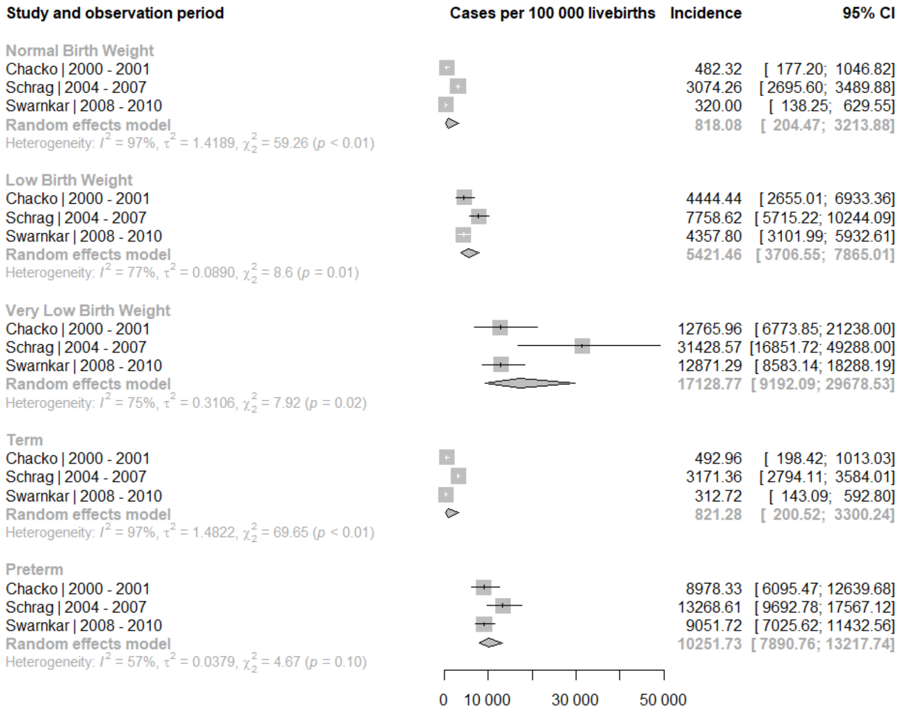

Abbreviations: **95% CI** 95% confidence interval

Supplement eFig. 2: Incidence of neonatal sepsis (early and late-onset sepsis combined) per 100 000 livebirths by setting

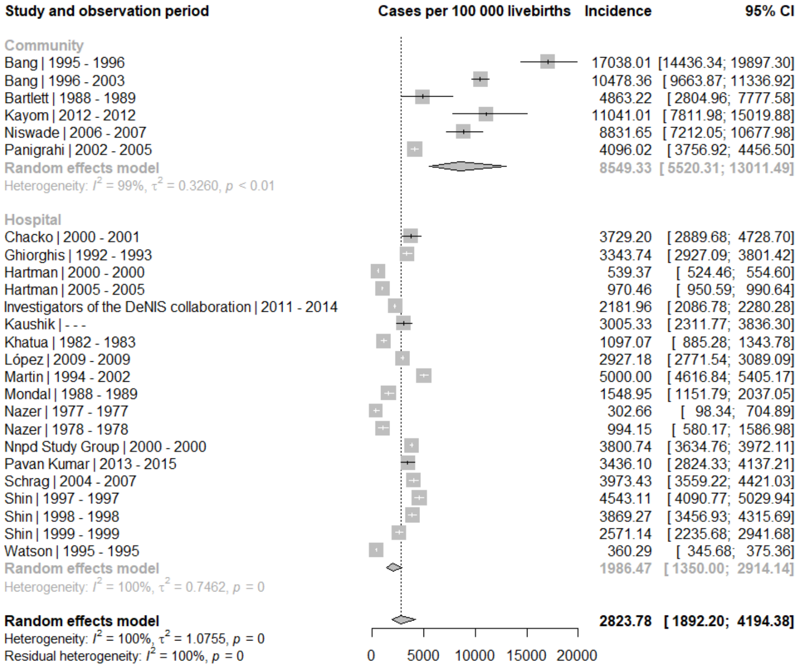

Abbreviations: **95% CI** 95% confidence interval

Supplement eFig. 3: Incidence of neonatal sepsis (early and late-onset sepsis combined) per 100 000 livebirths by income level

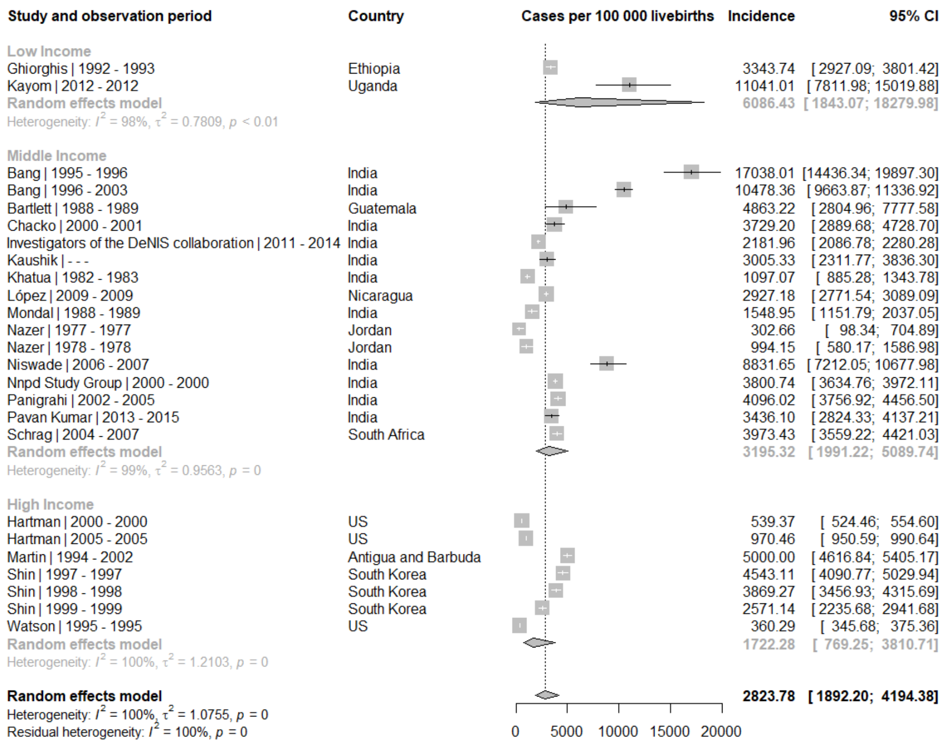

Abbreviations: **95% CI** 95% confidence interval

Supplement eFig. 4: Mortality of neonatal sepsis cases (early-onset sepsis, late-onset sepsis, and early and late-onset sepsis combined)

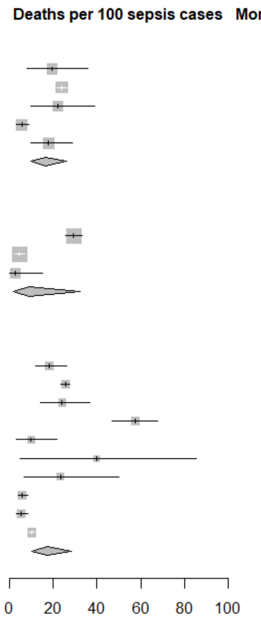

Study and observation period	Deceased cases	Sepsis cases	Mortality (%)	95% CI
**EOS**				
Chacko | 2000 - 2001	7	36	19.44	[8.19; 36.02]
Investigators of the DeNIS collaboration | 2011 - 2014	324	1351	23.98	[21.73; 26.35]
Panigrahi | 2002 - 2005	8	36	22.22	[10.12; 39.15]
Schrag | 2004 - 2007	17	289	5.88	[3.46; 9.25]
Swarnkar | 2008 - 2010	13	72	18.06	[9.98; 28.89]
Random effects model			16.35	[9.75; 26.13]
Heterogeneity: $I^2 = 90\%$, $\tau^2 = 0.3668$, $p < 0.01$				
**LOS**				
Investigators of the DeNIS collaboration | 2011 - 2014	172	583	29.50	[25.83; 33.39]
Panigrahi | 2002 - 2005	22	462	4.76	[3.01; 7.12]
Schrag | 2004 - 2007	1	34	2.94	[0.07; 15.33]
Random effects model			9.14	[2.06; 32.48]
Heterogeneity: $I^2 = 98\%$, $\tau^2 = 1.6452$, $p < 0.01$				
**EOS & LOS**				
Bang | 1995 - 1996	24	130	18.46	[12.20; 26.21]
Investigators of the DeNIS collaboration | 2011 - 2014	496	1934	25.65	[23.71; 27.65]
Kaushik | - - -	15	62	24.19	[14.22; 36.74]
Khatua | 1982 - 1983	53	92	57.61	[46.86; 67.85]
Mondal | 1988 - 1989	5	50	10.00	[3.33; 21.81]
Nazer | 1977 - 1977	2	5	40.00	[5.27; 85.34]
Nazer | 1978 - 1978	4	17	23.53	[6.81; 49.90]
Panigrahi | 2002 - 2005	30	498	6.02	[4.10; 8.49]
Schrag | 2004 - 2007	18	323	5.57	[3.34; 8.66]
Watson | 1995 - 1995	235	2280	10.31	[9.09; 11.63]
Random effects model			17.62	[10.25; 28.62]
Heterogeneity: $I^2 = 97\%$, $\tau^2 = 0.8994$, $p < 0.01$				

Abbreviations: **95% CI** 95% confidence interval, **EOS** early-onset sepsis, **LOS** late-onset sepsis

Supplement eFig. 5: Proportion of culture-proven sepsis (early-onset sepsis, late-onset sepsis, and early and late-onset sepsis combined)

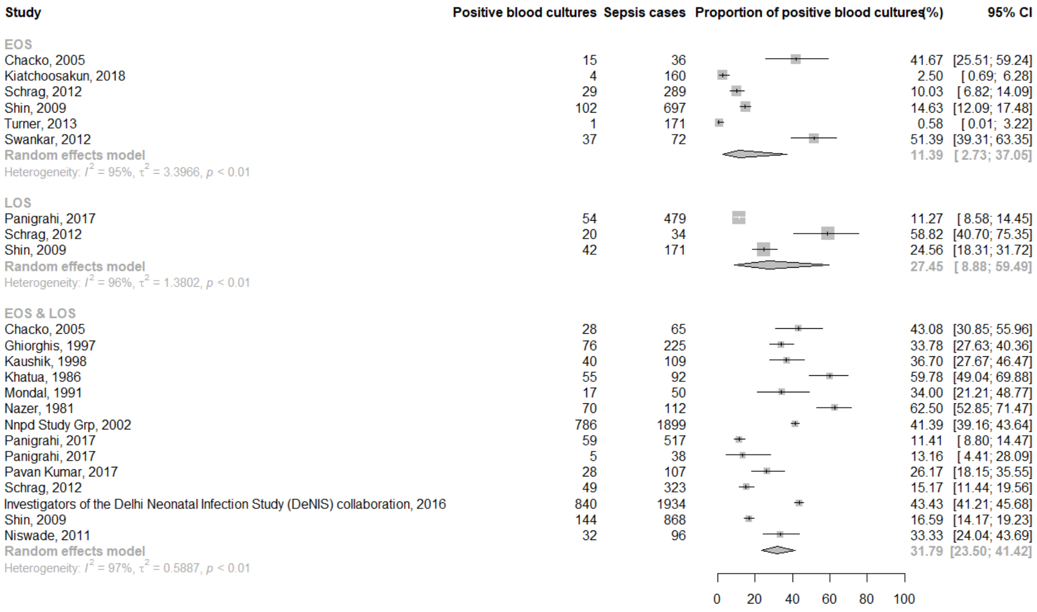

Supplement eTab. 1: Individual study characteristics

First author, publication year	Country, WHO region	Income level	Study period	Study design	Study setting and included patients	Sepsis case definition	Type of sepsis
**hospital-based**							
Chacko, 2005(18)	India,<br>South-East Asia Region	Middle	2000-2001	Single center<br>prospective<br>cohort study,<br>hospital records registry	Tertiary care teaching hospital, neonatal unit,<br>inborn,<br>no exclusion criteria stated,<br>subgroups: LBW and VLBW	Positive blood/cerebrospinal fluid cultures or developing clinical sepsis defined as any feature suggestive of sepsis such as altered body temperature, tachypnea/apnea, lethargy, poor feeding, shock or metabolic acidosis	EOS+LOS, EOS (≤72h), LOS (>72h)
Ghiorghis, 1997(19)	Ethiopia,<br>African Region	Low	1992-1993	Single center<br>retrospective<br>cohort study,<br>hospital records registry	Referral children's hospital, neonatal unit,<br>inborn,<br>no exclusion criteria stated	Clinical manifestations and/or positive blood culture (definition according to Alojipan & Andrews 1975(56): "Clinical manifestations considered for infants with infection included cyanosis, tachypnea, retractions, apnea, tachycardia, hypo- or hyperthermia, lethargy, irritability, seizures, feeding problems, vomiting, diarrhea, abdominal distention, jaundice, hepatosplenomegaly, and weight loss.")	EOS+LOS
Goulart, 2006(20)	Brazil,<br>Region of the Americas	Middle	2004	Single center<br>prospective<br>case-control study,<br>active observation	Referral hospital, NICU,<br>no exclusion criteria stated	Definition according to Goldstein consensus criteria(11)<br>Sepsis: SIRS in the presence of or as a result of suspected or proven infection;<br>SIRS: presence of at least 2/4 criteria, one of which must be abnormal temperature or leukocyte count:	EOS (≤72h)

						- Corebody temperature of 38.5 °C or 36 °C<br><br>- Tachycardia, defined as a mean heart rate > 2 SD above normal for age OR bradycardia OR otherwise unexplained persistent depression over a 0.5-hour time period<br><br>- Mean respiratory rate > 2 SD above normal for age or mechanical ventilation for an acute process<br><br>- Leukocyte count elevated or depressed for age or > 10% immature neutrophils;<br><br>Infection: suspected or proven (by positive culture, tissue stain, or polymerase chain reaction test) infection caused by any pathogen OR clinical syndrome associated with a high probability of infection;	
Hartman, 2013(21)	USA,<br><br>Region of the Americas	High	2000<br><br>2005	Multicenter<br><br>retrospective<br><br>cohort study,<br><br>hospital records registry	All non-federal hospitals in 7 states representative of the US,<br><br>Inborn and outborn,<br><br>no exclusion criteria stated	ICD-based implicit case identification (ICD-9–CM codes for both infection and acute organ failure)	EOS+LOS
Investigators	India,	Middle	2011-	Multicenter	3 Level III-care centres with	Culture-positive sepsis:	EOS+LO

of the Delhi Neonatal Infection Study (DeNIS) collaboration, 2016(22)	South-East Asia Region		2014	prospective<br><br>cohort study,<br><br>active observation	NICUs,<br><br>inborn,<br><br>exclusion criteria: Neonates requiring rehospitalisation after initial discharge	Isolation of a recognised pathogen from blood, cerebrospinal fluid, or other body fluids in neonates suspected to have sepsis on the basis of clinical features or maternal or perinatal risk factors, along with treatment involving appropriate type and duration of antibiotic therapy.<br>Cases of sepsis with positive culture for coagulase-negative staphylococci were labelled only if the clinical course was suggestive of sepsis and appropriate antibiotic therapy was given.<br><br>Culture-negative sepsis:<br><br>Baby has ALL of the following:<br><br>1.Any one of the clinical sign/symptoms: Difficulty feeding, convulsions, movement only when stimulated, watery stools, pus from umbilical stump, discharge (purulent) from ear, temperature (>37.5 °C or <36.5 °C), heart rate (>180 min or <100 min), respiratory rate (>60 min), severe chest in-drawing, grunting, apnea, capillary refill time > 3 sec, cyanosis, lethargy/drowsiness, bulging fontanel, abdominal distension, multiple (>10) skin pustules,c linicians' discretion<br><br>OR<br><br>Existence of predisposing risk factors: maternal fever within 7 days before delivery or foul smelling liquor or prolonged rupture of membranes (>18 h)	S, EOS (≤72h), LOS (>72h)

						OR<br><br>Radiological evidence of pneumonia<br><br>OR<br><br>Positive septic screen<br><br>2.Blood culture not done or no organisms detected in blood<br><br>3.Physician institutes appropriate treatment for sepsis	
Kaushik, 1998(23)	India,<br><br>South-East Asia Region	Middle	Not stated	Cohort study	Inborn,<br><br>no exclusion criteria stated,<br><br>subgroups: NBW, LBW	Clinical picture suggestive of septicaemia (definition according to Klein et al. 1990:(57) lethargy; mottled, pale skin; change in feeding pattern; vomiting)	EOS+LOS
Khatua, 1986(24)	India,<br><br>South-East Asia Region	Middle	1982-1983	Single center<br><br>prospective<br><br>cohort study,<br><br>active observation	Calcutta Medical College, nursery,<br><br>no exclusion criteria stated	Clinical manifestation and/or positive blood culture (table IV: refusal of feeds; lethargy; diarrhea; temperature change; abdominal distension; jaundice; vomiting; respiratory distress; sclerema; apneic spells; convulsion; hepatomegaly; splenomegaly)	EOS+LOS
Kiatchoosakun, 2019(25)	Thailand,<br><br>South-East Asia Region	Middle	2012-2013	Multicenter<br><br>retrospective<br><br>cohort study,<br><br>hospital records registry	3 Level II- and III-care hospitals: one university hospital, one regional hospital, one provincial hospital, NICUs,<br><br>inborn,<br><br>no exclusion criteria stated	Three or more clinical signs or laboratory results consistent with EOS, and further received antibiotics for at least 5 consecutive days.<br><br>Clinical signs or laboratory results that suggested sepsis: (1) increased oxygen, requirement or ventilatory support, (2) increase in apneic or bradycardic episodes or tachycardia, (3) hypotension or	EOS (≤72h)

						prolonged capillary refill time, (4) lethargy, (5) temperature instability, (6) ileus/feeding intolerance or abdominal distension, (7) glucose intolerance, and (8) base deficit >10 mmol/L	
López, 2013(26)	Nicaragua,<br><br>Region of the Americas	Middle	2009	Multicenter<br><br>prospective<br><br>trial,<br><br>active observation	18 "health care centers", one of which a specialized, level III-care university hospital,<br><br>no exclusion criteria stated	Clinical signs for neonatal sepsis: difficulty feeding; convulsions; hyperreactive; respiratory rate >60/min; subcostal indrawing; axillary temperature > 37.5C or < 35.5C (definition according to Carlin et al. 2008)(58)	EOS+LOS
Martin, 2007(27)	Antigua and Barbuda,<br><br>Region of the Americas	High	1994-2002	Single center<br><br>retrospective<br><br>cohort study,<br><br>hospital records registry	The "only full service hospital" in the country, special care nursery,<br><br>no exclusion criteria stated	Clinical features suggestive for sepsis (respiratory distress; fever, lethargy; poor feeding)	EOS+LOS
Mondal, 1991(28)	India,<br><br>South-East Asia Region	Middle	1988-1989	Single center<br><br>prospective<br><br>cohort study,<br><br>active observation	Referral hospital,<br><br>inborn,<br><br>no exclusion criteria stated	Clinical criteria (poor activity; refusal of feeds; hypothermia; respiratory distress; abdominal distension; hepatosplenomegaly; hyperthermia; sclerema; seizures; jaundice; bronchopneumonia; bleeding manifestation; arthritis; shock)<br>and/or positive blood culture<br><br>The total and differential white blood cell count, platelet, band count and micro-ESR were estimated in all cases [...] The neonates whose subsequent course and investigations did not reveal septicaemia were excluded from the study	EOS+LOS
Nazer,	Jordan,	Middle	1977	Single center	550-bedded university hospital,	Clinical criteria (definition according to	EOS+LO

1981(29)	Eastern Mediterranean Region		1978	prospective<br><br>cohort study,<br><br>active observation	NICU,<br><br>inborn,<br><br>no exclusion criteria stated	Behrman 1975)(59)	S
NNPD Study Group, 2002(30)	India,<br><br>South-East Asia Region	Middle	2000	Multicenter<br><br>prospective<br><br>cohort study	16 centers participating in the NNPD study group in India,<br><br>no exclusion criteria stated	Definition according to National Neonatal-Perinatal Database (60):<br><br>Septicemia (systemic bacterial infection) culture negative (clinical): In an infant having clinical picture suggestive of septicemia, the presence of any one of the following criteria is enough for assigning probable diagnosis of infection:<br><br>- Existence of predisposing factors: maternal fever or foul smelling liquor or prolonged rupture of membranes (>24 hrs) or gastric polymorphs (>5 per high power field)<br><br>- Positive septic screen (two of the four parameters (TLC <5000/mm, band to total polymorph ratio of > 0.2, ANC<1800/mm^3, CRP >1mg/dl, micro ESR>10mm 1st hour)<br><br>- Radiological evidences of pneumonia	EOS+LOS
Pavan Kumar, 2017(31)	India,<br><br>South-East Asia Region	Middle	2013-2015	Single center<br><br>prospective<br><br>cohort study,<br><br>active observation	Level II-care rural hospital, 10-bedded Neonatal Intensive Care Unit (NICU),<br><br>Inborn,<br><br>exclusion criteria: gross congenital anomalies	Young Infant Study Algorithm standard definitions of sepsis, adapted from National Healthcare Safety Network which included tachypnea, respiratory distress, prolonged capillary refill time, abnormal color, abdominal distension, poor sucking, irritability, convulsions, temperature abnormalities, lethargy, or apnea and/or positive blood culture	EOS+LOS

Schrag, 2012(32)	South Africa,<br><br>African Region	Middle	2004-2007	Single center<br><br>prospective<br><br>trial,<br><br>active observation	Level II - III care academic hospital,<br><br>inborn,<br><br>exclusion criteria: planned cesarean section, intepartum hemorrhage, known severe congenital malformation, intrauterine death confirmed before randomization, known allergy to chlorhexidine, face presentation, significant genital warts or ulcers, full cervical dilatation at randomization and age <15 years;<br><br>subgroups: preterm, VLBW, LBW;	Culture confirmed or clinical sepsis<br><br>EOS (clinical): A neonate hospitalised on days 0-2 of life and who in the absence of another recognizable congenital infection had at least one laboratory criteria and either: respiratory distress (one criterion required) or at least two clinical criteria<br><br>LOS (clinical): A neonate hospitalised between day three and 28 of life with at least one laboratory criteria and either: respiratory distress (two criteria required), OR one feature of respiratory distress and one other clinical criterion OR at least two other clinical criteria. Clinical criteria: respiratory distress; hypertension; pyrexia or hypothermia; abdominal/ feeding problems; bleeding diathesis, lethargy or irritability; central nervous system<br><br>Laboratory criteria :TLC; ANC; platelet count; CRP; elevated CSF white blood cell count (for details see: Supplemental Digital Content 1)	EOS+LOS, EOS (≤48h), LOS (>48h)
Shin, 2009(33)	South Korea, Western Pacific Region	High	1997<br><br>1998<br><br>1999	Multicenter<br><br>retrospective<br><br>cohort study,<br><br>hospital records registry	4 Level II - III care hospitals,<br><br>inborn and outborn,<br><br>no exclusion criteria stated	Neonatal sepsis was defi ned as a clinical syndrome characterized by systemic signs of infection and/or accompanied by bacteremia<br><br>Clinical sepsis was diagnosed when the doctor suspected it to be sepsis based on systemic<br><br>symptoms and signs, such as temperature instability, lethargy, apnea, poor feeding,<br><br>and respiratory or gastrointestinal disease (e.g. tachypnea and cyanosis or vomiting,	EOS+LOS

						diarrhea and abdominal distention), serology and/or radiology; TLC >30 000 cells/ L or <5000 cells/ L, CRP >1.0 mg/dL, risk factors for vertical transmission and/or intrapartum administration of antibiotics, and negative culture	
Swarnkar, 2012(34)	India,<br>South-East Asia Region	Middle	2008-2010	Single center<br>retrospective<br>cohort study,<br>hospital records registry	Rural hospital,<br>no exclusion criteria stated	Neonates who were clinically suspected to have bacterial infections within the first 48 hours of life, based on the risk factors and/or clinical features, were subjected to various hematological screening parameters and blood cultures (buffy coat smear examination, CRP, micro-ESR , TLC, ANC, and Immature (band cells) count / Total neutrophil count ratio)	EOS (≤48h)
Turner, 2013(35)	Thailand/ Myanmar,<br>South-East Asia Region	Middle	2009-2012	Single center<br>prospective<br>cohort study,<br>active observation	Health center for malaria in a refugee camp, special baby care unit,<br>inborn and outborn,<br>exclusion criteria: severe congenital abnormality identified prenatally or at birth, or had received antibiotics within the early neonatal period (≤6 days of age)	Fever (>38°C on one occasion or >37.5°C on two occasions separated by at least one hour) or at least two clinical features (poor perfusion, respiratory distress, persisting glucose imbalance, abdominal distension, bilious aspirates, or blood in the stool in a baby <72 hours of age)	EOS (<7d)
Watson, 2003(36)	USA,<br>Region of the Americas	High	1995	Multicenter<br>retrospective<br>cohort study,<br>hospital records registry	All non-federal hospitals in several states, representative of the US,<br>Inborn and outborn<br>no exclusion criteria stated	ICD-based implicit case identification (ICD-9–CM codes for both infection and acute organ failure)	EOS+LOS

community-based							
Bang, 2001(37)	India,<br><br>South-East Asia Region	Middle	1995-1996	Prospective<br><br>cohort study	Inborn and outborn<br><br>no exclusion criteria stated	Simultaneous presence of any two of the following six criteria any time during 0-28 days:<br>1. Baby which cried well at birth, it's cry became weak or abnormal, or stopped crying; or baby who earlier sucked or licked well, stopped sucking or mother feels that sucking became weak or reduced: or baby who was earlier conscious and alert, became drowsy or unconscious;<br>2. Skin temperature >99ºF or <95ºF;<br>3. Sepsis in skin or umbilicus;<br>4. Diarrhea or persistent vomiting or distension of abdomen;<br>5. Grunt or severe chest indrawing;<br>6. Respiratory rate ≥ 60 / minute even on counting twice	EOS+LOS
Bang, 2005(38)	India,<br><br>South-East Asia Region	Middle	1996-2003	Trial,<br><br>active observation	Home-based surveillance by trained village health workers in rural communities in peripheral India,<br><br>no exclusion criteria stated	Simultaneous presence of any two or more criteria in a neonate denoted sepsis :<br><br>A. Criteria used in 1995 to 1998:<br><br>1. Previously normal cry became weak/stopped or previously normal baby became drowsy/unconscious or previously normal sucking became weak or stopped.<br>2. Baby cold to touch or fever (skin temperature >99°F)<br>3. Skin infection or umbilical infection<br>4. Vomiting or diarrhea or abdominal distension<br>5. Respiratory rate ≥60<br>6. Grunt or chest indrawing<br><br>B. Criteria used in 1998 to 2003:	EOS+LOS

						1. Previously normal cry became weak/stopped<br>2. Previously normal baby became drowsy/unconscious<br>3. Previously normal sucking became weak/stopped<br>4. Baby cold to touch or fever (>99°F)<br>5. Skin infection or umbilical infection<br>6. Abdominal distension or vomiting<br>7. Grunt or chest indrawing	
Bartlett, 1991(39)	Guatemala,<br><br>Region of the Americas	Middle	1988-1989	Prospective and retrospective<br><br>cohort study	No exclusion criteria stated	Clinical diagnoses were assigned by the study physicians based on history and physical examination […] in most cases without laboratory examination	EOS+LOS
Kayom, 2018(40)	Uganda,<br><br>African Region	Low	2012	Prospective and retrospective<br><br>cohort study	Community based surveillance, referral of septic neonates to the emergency unit of the national referral hospital;<br><br>inborn and outborn,<br><br>exclusion criteria: gross congenital malformation and extremely low birthweight	WHO IMNCI criteria(61):<br>Neonate that had temperature > 37.5°C or felt hot to touch, convulsions (by history), fast breathing (> 60 breaths/minute), severe chest indrawing, nasal flaring, grunting, bulging fontanelle, pus draining from ear, umbilical redness extending to the skin, feels cold (by history), many or severe skin pustules, difficult to wake up, cannot be calmed within 1 h, less than normal movement, not able to feed and not able to attach to breast or suck	EOS+LOS
Niswade, 2011(41)	India,<br><br>South-East Asia Region	Middle	2006-2007	Prospective<br><br>cohort study	No exclusion criteria stated	Presumption of diagnosis based on the typical history of illness	EOS+LOS
Panigrahi, 2017(42)	India,<br><br>South-East Asia Region	Middle	2002-2005	Prospective<br><br>cohort study	223 villages in rural India,<br><br>inborn and outborn,<br><br>exclusion criteria: >60 days old,	Clinical evaluation included specific questions and physical examination on the 12 signs/symptoms of sepsis adapted from the IMCI guidelines (62)	EOS+LOS (≤28d), EOS (≤72h), LOS

					congenital anomaly, not given consent	Only cases of clinical sepsis confirmed by a study paediatrician were included. Clinical sepsis included negative blood/CSF cultures or respiratory symptoms (pneumonia), when the infant was treated with antibiotics for 5 or more days or died within 5 days of enrolment.	(>72h≤28 d)
Raihana, 2017(43)	Bangladesh,<br><br>South-East Asia Region	Middle	2013-2015	Prospective<br><br>trial	Inborn and outborn,<br><br>exclusion criteria: women whose breastfeeding status was unknown or missing	Clinical sepsis definition according to “The Young-Infants Clinical Science Study Group”	EOS (<7d)

Abbreviations: **ANC** absolute neutrophil count, **CRP** C-reactive protein, **CSF** cerebrospinal fluid, **EOS** early-onset sepsis, **ESR** erythrocyte sedimentation rate, **ICD** International Statistical Classification of Diseases and Related Health Problems, **ICD-CM** International Statistical Classification of Diseases and Related Health Problems Clinical Modification, **IMCI** Integrated Management of Childhood Illness, **LOS** late-onset sepsis, **NBW/LBW/VLBW** normal/low/ very low birth weight, **NICU** neonatal intensive care unit, **NNPD** National Neonatal-Perinatal Database, **SD** standard deviation, **SIRS** systemic inflammatory response syndrome, **TLC** total leucocyte count, **WHO** World Health Organisation

Supplement eTab. 2: Risk of bias of the included studies

Item	1.	2.	3.	4.	5.	6.	7.	8.	9.	10.	11. Summary item
Hartman, 2013	1	1	1	1	1	1	3	1	1	1	2
Shin, 2009	3	1	1	1	1	1	3	1	1	1	3
Watson, 2003	1	1	1	1	1	1	3	1	1	1	2
Turner, 2013	3	1	1	1	1	1	3	1	1	1	3
Niswade, 2011	3	1	1	1	1	3	3	1	1	1	3
Bang, 2001	3	1	1	1	1	1	3	1	1	1	3
Swarnkar, 2012	3	1	1	1	1	3	3	1	1	1	3
Bartlett, 1991	3	1	1	1	1	3	3	1	1	1	3
Bang, 2005	3	1	1	1	1	1	3	3	1	1	3
Chacko, 2005	3	1	1	1	1	3	3	1	1	1	3
Ghiorghis , 1997	3	1	1	1	1	3	3	1	1	1	3
Goulart, 2006	3	1	1	1	1	1	1	1	1	1	2
DeNIS investigators, 2016	3	1	1	1	1	3	3	1	1	1	3
Kaushik, 1998	3	1	1	1	1	3	3	1	1	1	3
Kayom, 2018	3	1	1	3	1	1	1	1	1	1	3
Khatua, 1986	3	1	1	1	1	3	3	1	1	1	3
Kiatchoosakun, 2019	3	1	1	1	1	1	3	1	1	1	3
López, 2013	3	1	1	1	1	3	3	1	1	1	3
Martin, 2007	1	1	1	1	1	3	3	1	1	1	2

Mondal, 1991	3	1	1	1	1	3	3	1	1	1	3
Nazer, 1981	3	1	1	1	1	3	3	1	1	1	3
Nnpd Study Grp, 2002	3	1	1	1	1	3	3	1	1	1	3
Panigrahi, 2017	3	1	1	3	1	1	1	1	1	1	3
Pavan Kumar, 2017	3	1	1	1	1	1	1	1	1	1	2
Raihana, 2017	3	3	1	3	1	1	1	1	1	1	3
Schrag, 2012	3	3	1	1	1	1	3	1	1	1	3

Hoy Risk of Bias Assessment

1. Was the study's target population a close representation of the national population in relation to relevant variables? (1=low/3=high risk of bias)

2. Was the sampling frame a true or close representation of the target population? (1=low/3=high risk of bias)

3. Was some form of random selection used to select the sample, OR was a census undertaken? (1=low/3=high risk of bias)

4. Was the likelihood of nonresponse bias minimal? (1=low/3=high risk of bias)

5. Were data collected directly from the subjects (as opposed to a proxy)? (1=low/3=high risk of bias)

6. Was an acceptable case definition used in the study? (1=low/3=high risk of bias)

7. Was the study instrument that measured the parameter of interest shown to have validity and reliability? (1=low/3=high risk of bias)

8. Was the same mode of data collection used for all subjects? (1=low/3=high risk of bias)

9. Was the length of the shortest prevalence period for the parameter of interest appropriate? (1=low/3=high risk of bias)

10. Were the numerator(s) and denominator(s) for the parameter of interest appropriate? (1=low/3=high risk of bias)

11. Summary item on the overall risk of study bias (1=low/2=moderate/3=high risk of bias)

Supplement eTab. 3: Underlying pathogens by sepsis type, 2000-2019

First author, publication year	Number of isolates	Gram-positive bacteria	Gram-negative bacteria	Funghi	Antimicrobial resistance
**EOS & LOS**					
Nnpd Study Group, 2002	909	*Staphylococcus aureus* 17·5%<br>*Staphylococcus albus* 4·9%	*Klebsiella pneumoniae* 31·2%<br>*E coli* 10·5%<br>*Pseudomonas* spp 6·8%<br>*Acinetobacter* spp, 4·9%	*Candida* spp. 8·7%	*Klebsiella penumoniae* non-susceptible to: amikacin 35·6%, ciprofloxacin 26·7%, ceftazidime 34·4%, gentamicin 78·6%, cefotaxime 78·5%;<br>*Staphylococcus aureus* non-susceptible to: vancomycin 26·1%, amikacin 43·3%, cefotaxime 46·8%, penicillin 89·3%, ampicillin 91·7%
Pavan Kumar, 2017	28	*Staphylococcus aureus* 35·7%<br>Nonhemolytic streptococci 3·5%	*Klebsiella* spp . 21·4%<br>Untyped gram-negatives 21·4%<br>*E coli* 10·7%<br>*Pseudomonas aeruginosa* 3·5%<br>Non-fermenting gram-negatives 3·5%		Gram-negative bacteria resistant to: ampicillin 100%, cloxacillin 64·7%, gentamicin 52·9%, piperacillin 47·1%, linezolid 41·2%, ceftriaxone/cefotaxime 31·2%, amikacin 17·6%, ciprofloxacin 11·8%, meropenem 11·8%;<br>*Staphylococcus aureus* resistant to: ampicillin 90%, cloxacillin 90%, piperacillin 30%, gentamicin 20%, ceftriaxone/cefotaxime 20%, ciprofloxacin 20%, linezolid 0%, amikacin 0%, meropenem 0%
Investigators of the Delhi Neonatal Infection Study (DeNIS) collaboration,	1005	Coagulase-negative staphylococci 14·9%	*Acinetobacter* spp, 22·1%<br>*Klebsiella* spp. 16·8%	*Candida* spp. 0·7%	Gram-negative bacteria:Amikacin resistance: *Acinetobacter* spp, 84%, *Klebsiella* spp. 45%, *E coli* 23%, *Pseudomonas* spp 17%, *Enterobacter* spp 36%

2016		*Staphylococcus aureus* 12·1%<br>*Enterococcus* spp. 5·6%<br>*Streptococcus* spp. 1·2%<br>Group B streptococci 0·8%<br>Others 1·0%	*E coli* 13·6%<br>*Pseudomonas* spp 6·8%<br>*Enterobacter* spp 4·4%		Meropenem resistance: *Acinetobacter* spp, 81%, *Klebsiella* spp. 36%, *E coli* 14%, *Pseudomonas* spp 27%, *Enterobacter* spp 17%<br>Colistin resistance: 1% of gram-negative isolates.<br>Gram-positive bacteria:<br>Amikacin resistance: coagulase-negative staphylococci 14%, *Staphylococcus aureus* 2%<br>Methicillin resistance: coagulase-negative staphylococci 60%, *Staphylococcus aureus* 37%.<br>Vancomycin resistance: *Enterococcus* spp 26%, coagulase-negative staphylococci 0%, *Staphylococcus aureus 0%*
Niswade, 2011	32	*Streptococcus pneumoniae* 25·0%<br>*Staphylococcus aureus* 25·0%	*Klebsiella* spp 21·9%<br>*E coli* 28·1%		
**EOS**					
Chacko, 2005	15	*Staphylococcus aureus* 13·3%<br>*Streptococcus viridans* 7%	*Pseudomonas* spp 60%,<br>*Klebsiella pneumoniae* 13·3%<br>*E coli* 7%		
Kiatchoosakun, 2018	4	*Streptococcus agalactiae* 50%	*E coli* 25%		

			Pseudomonas spp 25%		
Turner, 2013	1		*E coli* 100%		
Swarnkar, 2012	37	Gram-positive organisms 43%<br>*Staphylococcus aureus* 38%<br>other staphylococci 5·4%	Gram-negative organisms 56·75%<br>*Klebsiella pneumoniae* 48·6%<br>*E coli* 13·5%		
Schrag, 2012	29	Group B streptococci 55·2%<br>*Enterococcus* faecalis 10·3%<br>*Staphylococcus aureus* 6·9%<br>*Streptococcus viridans* 6·9%	*E coli* 6·9%<br>*Acinetobacter baumannii* 6·9%<br>*Klebsiella pneumoniae* 3·4%<br>*Acinetobacter lwoffii* 3·4%		
Panigrahi, 2017	5		*Klebsiella* spp 40·0%<br>*E coli* 20·0%<br>other gram-negatives 40·0%		
**LOS**					
Panigrahi, 2017	54	*Staphylococcus aureus* 20·3%<br>other gram-positives 1·8%	*Klebsiella* spp 50·0%<br>*E coli* 12·3%<br>other gram-negatives 9·3%	*Candida* spp. 1·8%	
Schrag, 2012	20	Group B streptococci 25%<br>*Enterococcus faecium* 10%	*E coli* 40%<br>*Klebsiella* spp 10%		

		Staphylococcus aureus 10%<br><br>*Streptococcus* species 5%			

Abbreviations: **EOS** early-onset sepsis, **LOS** late-onset sepsis, ***E coli*** *Escherichia coli*
