# Supplementary material for: Global incidence and mortality of neonatal sepsis: a systematic review and meta-analysis
Source: Arch Dis Child. 2021 Jan 22;106(8):745–52. doi: 10.1136/archdischild-2020-320217 (PMC8311109; doi:10.1136/archdischild-2020-320217)
Supplement: Supplementary data [file archdischild-2020-320217supp001.pdf]

```
#==============
# LOAD PACKAGES
#==============
install.packages("meta")
library(meta)

###labels
install.packages("expss")
library("expss")

setwd("~/Sepsis_Review/Analyse")

#=============
## READ DATA
#=============
library(readxl)
Neonates<- read_excel("C:/Users/ReichertF/Documents/Sepsis_Review/Analyse/
Neonates_Analysetab_2020_01_09.xlsx")

#============
## DATA PREP
#============
###Subset of studies of 1st SR
Test<- subset(Neonates, Test==1)

###by sepsis type
####Label type_sepsis: 1 EOS; 2 LOS; 3 mixed
EOS<- subset(Neonates,(type_sepsis==1 & subgroup==0))
LOS<- subset(Neonates,(type_sepsis==2 & subgroup==0))
mixed<- subset(Neonates,(type_sepsis==3 & subgroup==0))
all<- subset(Neonates,subgroup==0)
all$type_sepsis<-factor(all$type_sepsis, levels = c(1,2,3), labels =
c("EOS","LOS","EOS & LOS"))

###by subgroup
#####Label subgroup: 0 none 1 NBW; 2 LBW ; 3 VLBW; 4 preterm;  5 term; 9 LBW
(incl.VLBW)
mixed_subgroup<- subset(Neonates,(type_sepsis==3 & subgroup!=0))
#subgroups (GA+BW), no study with subgroup "9"
EOS_subgroup<- subset(Neonates,(type_sepsis==1 & subgroup!=0 & subgroup!=9))
#subgroups (GA+BW), without overlapping defintion for LBW ("9")
LOS_subgroup<- subset(Neonates,(type_sepsis==2 & subgroup!=0 & subgroup!=9))
#subgroups (GA+BW), without overlapping defintion for LBW ("9")
#mixed_GA<- subset(Neonates,(type_sepsis==3 & (subgroup==4 | subgroup==5)))
#by gestational age
#EOS_GA<- subset(Neonates,(type_sepsis==1 & (subgroup==4 | subgroup==5)))
#by gestational age
#mixed_GG<- subset(Neonates,(type_sepsis==3 & (subgroup==1 | subgroup==2 |
subgroup==3)))   #by birth weight
#EOS_GG<- subset(Neonates,(type_sepsis==1 & (subgroup==1 | subgroup==2 |
subgroup==3)))     #by birth weight
#mixed_GGrisk<- subset(Neonates,(type_sepsis==3 & (subgroup==2 | subgroup==3)))
#only risk groups birth weight
#EOS_GGrisk<- subset(Neonates,(type_sepsis==1 & (subgroup==2 | subgroup==3)))
#only risk groups birth weight
#mixed_all<- subset(Neonates,type_sepsis==3)
```

```
EOS$decade[EOS$year_from >=1989 & EOS$year_from<=1998]<-2
EOS$decade[EOS$year_from >=1999 & EOS$year_from<=2008]<-3
EOS$decade[EOS$year_from >=2009]<-4
EOS$decade <- factor(EOS$decade, levels = c(1,2,3,4), labels = c("Before 1989",
"1989-1998", "1999-2008", "2009-2018"))

LOS$decade<-9
LOS$decade[LOS$year_from <=1988]<-1
LOS$decade[LOS$year_from >=1989 & LOS$year_from<=1998]<-2
LOS$decade[LOS$year_from >=1999 & LOS$year_from<=2008]<-3
LOS$decade[LOS$year_from >=2009]<-4
LOS$decade <- factor(LOS$decade, levels = c(1,2,3,4), labels = c("Before 1989",
"1989-1998", "1999-2008", "2009-2018"))

mixed$decade<-9
mixed$decade[mixed$year_from <=1988]<-1
mixed$decade[mixed$year_from >=1989 & mixed$year_from<=1998]<-2
mixed$decade[mixed$author =="Kaushik"]<-2 #Kaushik: Publikation 1998
mixed$decade[mixed$year_from >=1999 & mixed$year_from<=2008]<-3
mixed$decade[mixed$year_from >=2009]<-4
mixed$decade <- factor(mixed$decade, levels = c(1,2,3,4), labels = c("Before
1989", "1989-1998", "1999-2008", "2009-2018"))

### by setting: 0 community; 1 hospital
mixed$setting<- factor(mixed$setting, levels = c(0,1), labels = c("Community",
"Hospital"))
var_lab(mixed$setting) = "Study setting"
EOS$setting<- factor(EOS$setting, levels = c(0,1), labels = c("Community",
"Hospital"))

### by WHO region
####Label: 1=African Region, 2=Region of the Americas, 3=South-East Asia Region,
4= European Region,5= Eastern Mediterranean Region, 6=Western Pacific Region
mixed$Region<- factor(mixed$WHO_region, levels = c(1,2,3,4,5,6), labels =
c("AFR", "PAR", "SEAR", "EUR", "EMR", "WPR"))
EOS$Region<- factor(EOS$WHO_region, levels = c(1,2,3,4,5,6), labels = c("AFR",
"PAR", "SEAR", "EUR", "EMR", "WPR"))

### by income
####Label: 1=low 3=middle 4=high
mixed$Income_Group<- factor(mixed$income2, levels = c(1,3,4), labels = c("Low
Income", "Middle Income", "High Income"))
EOS$Income_Group<- factor(EOS$income2, levels = c(1,3,4), labels = c("Low
Income", "Middle Income", "High Income"))


#=========================
## meta-analysis, Incidence
#=========================
###Test - reproducton of results of previous SR
Carolin<-
metaprop(n_sepsis_calc,n_livebirths,studlab=paste(author,year_from),method.tau="S[illegible]
= "inverse",data=Test,pscale = 100000)
forest(Carolin,leftcols=c("studlab"),rightcols=c("effect",
"ci"),pscale=100000,hetstat=F,comb.fixed=F,rightlabs=c("Incidence","95% CI"))
```

```
### time
####mixed
metamixed_decades<-
metaprop(n_sepsis_calc,n_livebirths,studlab=paste(author,year_from,"-",year_to),me
= "inverse",data=mixed,pscale = 100000,byvar = decade)
forest(metamixed_decades,leftcols=c("studlab"),leftlabs=c("Study and observation
period"),smlab="Cases per 100 000 livebirths",print.byvar=FALSE,
       rightcols=c("effect",
"ci"),xlim=c(0,20000),pscale=100000,hetstat=T,comb.fixed=F,rightlabs=c("Incidence
CI"))
####EOS
metaEOS_decades<-
metaprop(n_sepsis_calc,n_livebirths,studlab=paste(author,year_from,"-",year_to),me
= "inverse",data=EOS,pscale = 100000,byvar = decade)
forest(metaEOS_decades,leftcols=c("studlab"),leftlabs=c("Study and observation
period"),smlab="Cases per 100 000 livebirths",print.byvar=FALSE,
       rightcols=c("effect",
"ci"),xlim=c(0,20000),pscale=100000,hetstat=T,comb.fixed=F,rightlabs=c("Incidence
CI"))
####LOS
metaLOS_decades<-
metaprop(n_sepsis_calc,n_livebirths,studlab=paste(author,year_from,"-",year_to),me
= "inverse",data=LOS,pscale = 100000,byvar = decade)
forest(metaLOS_decades,leftcols=c("studlab"),leftlabs=c("Study and observation
period"),smlab="Cases per 100 000 livebirths",print.byvar=FALSE,
       rightcols=c("effect",
"ci"),xlim=c(0,20000),pscale=100000,hetstat=T,comb.fixed=F,rightlabs=c("Incidence
CI"))

###setting
####mixed
metamixed_setting<-
metaprop(n_sepsis_calc,n_livebirths,studlab=paste(author,"|",year_from,"-",year_to
= "inverse",data=mixed,pscale = 100000,byvar = setting)
forest(metamixed_setting,leftcols=c("studlab"),leftlabs=c("Study and observation
period"),
       colgap.forest.left="1cm",smlab="Cases per 100 000 livebirths",
       print.byvar = F,rightcols=c("effect",
"ci"),xlim=c(0,20000),pscale=100000,hetstat=T,comb.fixed=F,rightlabs=c("Incidence
CI"))
####EOS
metaEOS_setting<-
metaprop(n_sepsis_calc,n_livebirths,studlab=paste(author,"|",year_from,"-",year_to
= "inverse",data=EOS,pscale = 100000,byvar = setting)
forest(metaEOS_setting,leftcols=c("studlab"),leftlabs=c("Study and observation
period"),colgap.forest.left="1cm",smlab="Cases per 100 000
livebirths",print.byvar = F,rightcols=c("effect",
"ci"),xlim=c(0,50000),pscale=100000,hetstat=T,comb.fixed=F,rightlabs=c("Incidence
CI"))

###WHOregions
####mixed
metamixed_WHO<-
metaprop(n_sepsis_calc,n_livebirths,studlab=paste(author,"|",year_from,"-",year_to
= "inverse",data=mixed,pscale = 100000,byvar = Region)
forest(metamixed_WHO,leftcols=c("studlab", "country","setting"),leftlabs=c("Study
[illegible]
```

```
#forest(metamixed_GGrisk,leftcols=c("studlab"),rightcols=c("effect",
"ci"),xlim=c(0,50000),pscale=100000,hetstat=T,print.Q=T,comb.fixed=F,rightlabs=c("
CI"), colgap.forest.left = "60mm")
#metaEOS_GGrisk<-
metaprop(n_sepsis_calc,n_livebirths,studlab=paste(author,"|",year_from,"-",year_to
= "inverse",data=EOS_GGrisk,pscale = 100000,byvar = Birthweight)
#forest(metaEOS_GGrisk,leftcols=c("studlab"),leftlabs=c("Study and observation
period"),smlab="Cases per 100 000 livebirths",
#       print.byvar=FALSE,rightcols=c("effect",
"ci"),xlim=c(0,50000),pscale=100000,hetstat=T,print.Q=T,comb.fixed=F,rightlabs=c("
CI"), colgap.forest.left = "60mm")

#=====================
## mortality
#=====================
all_mort<-subset(all,n_deaths>0)
all_mort$n_deaths<-as.integer(all_mort$n_deaths)
metaall_mort<-
metaprop(n_deaths,n_mort_denom,studlab=paste(author,"|",year_from,"-",year_to),met
= "inverse",data=all_mort,pscale = 100, byvar = type_sepsis)
forest(metaall_mort,leftcols=c("studlab","n_deaths","n_mort_denom"),leftlabs=c("St
and observation period","Deceased cases", "Sepsis
cases"),digits.addcols.left=0,just.addcols.left = c("right"),
       colgap.forest.left="1cm",smlab="Deaths per 100 sepsis cases",
       print.byvar=FALSE,rightcols=c("effect",
"ci"),xlim=c(0,100),pscale=100,hetstat=T,comb.fixed=F,rightlabs=c("Mortality
(%)","95% CI"), overall = F,overall.hetstat = FALSE,)

#====================
## culture positivity
#===================
library(readxl)
#xblood_all1x <- read_excel("C:/Users/ReichertF/Documents/Sepsis_Review/NEONATES_
Pathogens_Resistances.xlsx",sheet ="all(1x)")
blood <- read_excel("C:/Users/ReichertF/Documents/Sepsis_Review/NEONATES_
Pathogens_Resistances.xlsx",sheet ="EOS+LOS BK")
#xblood_all1x = as.data.frame(blood_all1x)
blood = as.data.frame(blood)
blood$type_sspsis<-factor(blood$type_sspsis, levels = c(1,2,3), labels =
c("EOS","LOS","EOS & LOS"))

###by sepsis type
metablood<-
metaprop(n_culture_pos,n_culture,studlab=paste(Author),method.tau="SJ",method =
"inverse",data=blood,pscale = 100, byvar = type_sspsis)
forest(metablood,
       leftcols=c("studlab","n_culture_pos","n_culture"),leftlabs=c("Study",
"Positive blood cultures", "Sepsis
cases"),digits.addcols.left=0,just.addcols.left = c("right"),
       colgap.forest.left="1cm",smlab="Proportion of positive blood cultures",
       print.byvar=FALSE,rightcols=c("effect",
"ci"),xlim=c(0,100),pscale=100,hetstat=T,comb.fixed=F,rightlabs=c("(%)","95%
CI"), overall = F,overall.hetstat = FALSE,)

###overall
#xmetablood_all1x<-
```
